# Biomimetic Approaches for the Development of New Antifouling Solutions: Study of Incorporation of Macroalgae and Sponge Extracts for the Development of New Environmentally-Friendly Coatings

**DOI:** 10.3390/ijms20194863

**Published:** 2019-09-30

**Authors:** Ilse Sánchez-Lozano, Claudia Judith Hernández-Guerrero, Mauricio Muñoz-Ochoa, Claire Hellio

**Affiliations:** 1 Instituto Politécnico Nacional, Centro Interdisciplinario de Ciencias Marinas, Av. Instituto Politécnico Nacional S/N. Col. Playa Palo de Santa Rita, 23096 La Paz, Baja California Sur, Mexico; gaias.dreams@gmail.com (I.S.-L.); mmunozo@ipn.mx (M.M.-O.); 2 Univ Brest, CNRS, IRD, Ifremer, LEMAR, Institut Universitaire Européen de la Mer, F-29280 Plouzané, France

**Keywords:** adhesion, algae, antifouling, biofilm, extracts, field assays, sponges, *Haliclona caerulea*, *Laurencia gardneri*, *Sargassum horridum*, *Ircinia* sp.

## Abstract

Biofouling causes major economic losses in the maritime industry. In our site study, the Bay of La Paz (Gulf of California), biofouling on immersed structures is a major problem and is treated mostly with copper-based antifouling paints. Due to the known environmental effect of such treatments, the search for environmentally friendly alternatives in this zone of high biodiversity is a priority to ensure the conservation and protection of species. The aim of this work was to link chemical ecology to marine biotechnology: indeed, the natural defense of macroalgae and sponge was evaluated against biofoulers (biofilm and macrofoulers) from the same geographical zone, and some coatings formulation was done for field assays. Our approach combines in vitro and field bioassays to ensure the selection of the best AF agent prospects. The 1st step consisted of the selection of macroalgae (5 species) and sponges (2 species) with surfaces harboring a low level of colonizers; then extracts were prepared and assayed for toxicity against *Artemia*, activity towards key marine bacteria involved in biofilm formation in the Bay of La Paz, and the potency to inhibit adhesion of macroorganisms (phenoloxidase assays). The most active and non-toxic extracts were further studied for biofouling activity in the adhesion of the bacteria involved in biofilm formation and through incorporation in marine coatings which were immersed in La Paz Bay during 40 days. In vitro assays demonstrated that extracts of *Laurencia gardneri, Sargassum horridum* (macroalgae), *Haliclona caerulea* and *Ircinia* sp. (sponges) were the most promising. The field test results were of high interest as the best formulation were composed of extracts of *H. caerulea* and *S. horridum* and led to a reduction of 32% of biofouling compared with the control.

## 1. Introduction

In the marine ecosystem, many benthic organisms are likely to be colonized by other sessile organisms. This settlement process is denominated epibiosis or biofouling [1]. In order to regulate epibiosis, chemical defense systems have been developed by marine organisms [2]. Biofouling on man-made immersed structures causes an increase in the weight or volume leading to economic losses for many industrial sectors such as shipping, energy producing, fishing or aquaculture. In addition, biofilms have been proven to accelerate the corrosion process [3,4]. Therefore, the search for methods to reduce and delay the process of settlement and growth of bacteria and other organisms involved in the biofouling process is necessary. In previous years, paints formulated with Tributyltin (TBT) were used and provided over 10 years efficacy, however due to adverse environmental effects, its use has been banned. Currently, many antifouling paints formulations are based on copper and booster biocides [3,5], despite the fact that it has been proven that some of these compounds bring serious ecological problems [6].

The Gulf of California is an ecosystem with high priority for conservation. It is considered as one of the richest zone for biodiversity and thus includes several UNESCO World Heritage Sites. For these reasons, Captain Jacques Cousteau referred to this zone as “the world’s aquarium” [7]. Our research site is based in La Paz Bay (which is part of the Gulf of California), where most human marine activities are centered on tourism, fishing and aquaculture. The nautical tourism (yacht, mega yacht and sailing boats) has boomed in the area which now offers about 750 combs over 7 marinas, besides about 100 more boats that are permanently anchored in the bay (personal communication of the vice president of the Marine Tourism Association). Leaching of antifouling paints from all these boats add some significant pressure to the marine organisms within this pristine location.

In order to combat biofouling, while respecting the environment, a significant step forward would be to abandon molecules with a broad spectrum of activity in order to formulate paints with molecules having targeted activity only against colonizing species in a specific geographical area. This type of solution would be of course usable only for boat cruising in a single geographical area (which is the case of the majority of recreational boats and fishing boats and ferries). In order to achieve this, we decided first to design antifouling assays targeting the main colonizers found on immersed structures in the Bay of La Paz and thus not necessarily used the standard models species used for broad spectrum coatings.

Based on the fact that macroalgae and sponges synthetized metabolites to inhibit biofouling of their surfaces, such compounds could be used as antifouling (AF) agents to protect man-made immersed structures [8,9]. The idea is to use natural compounds with AF activity that can replace the biocidal chemical compounds commonly used in marine paints. Through this biomimetic-based approach, the purpose is to test the use of natural compounds with AF activity in order to avoid the use of marine paints containing excess biocides and environmental contamination in this area of high marine biodiversity. Moreover, the chances of success of discovering new AF agents increases when marine organisms from the same environment are evaluated [10]. This was corroborated by another study in which extracts of bacteria isolated from the coasts of La Paz Bay, B.C.S. presented high antimicrobial properties against 2 genera of bacteria involved in the biofouling process of the same environment [11].

Marine macroalgae produce a large number of compounds with AF activity, especially, extracts and compounds from Rhodophyceae have been shown to be particularly active. Regarding sponges, there has been a discovery of a high number of new compounds, which make this group one of the most interesting and it has been related to the association with symbiotic microorganisms [12].

Numerous studies aiming at the evaluation of AF activity are based on laboratory bioassays or on field tests (where extracts were incorporated in hydrogels or in paint) [13,14,15,16]. However, previous works highlighted that besides the laboratory tests, the extracts must be included in a paint matrix in order to carry out tests in the marine environment and the results do not always corroborate with the ones obtained from the laboratory assay [17]. Thus, both approaches must be carried out: laboratory assays in order to characterize the mode of action of the compound and fields tests in order to determine the range of action in various conditions of utilization and environments.

In the present study, the antifouling potential of a selection of 5 macroalgae and 2 sponges from the coast of Baja California Sur were evaluated with a different approach from other research. Instead of running extensive screening program in laboratories toward many fouling species, followed by toxicity tests and then immersion assays, we decided to take a more efficient approaches based on the following steps: 1) isolate and cultivate key bacteria involved in biofilm formation (5 Gram-positive and 6 Gram-negative); 2) select organisms with low epibiosis and produce some extracts; 3) evaluate the toxicity of these extracts and their capacity to inhibit both growth of the selected bacteria and the activity of phenoloxidase (this test is used as proxy for anti-barnacles and mussels activities as this enzyme is involved in the processes of the settlement, and is environmentally friendly as it avoid running assays on larvae); 4) only the best extracts (active against the bacteria, inhibit the activity of the phenoloxidase and non-toxic) are then further studied; 5) the selected extracts are evaluated for their potency at inhibiting the adhesion of the selected bacteria (this approach is original as many researchers focus only growth inhibition of model bacteria; the approach we take give priority to minimize the use of biocides, thus reducing the bacterial resistance and ensuring the protection of the environment [18]; 6) the most promising extracts are then included in experimental painting matrix and immersed in the sea; 7) extracts leading to best results in the immersion test are then further tested in laboratory to determine the mode of action and to determine which extracts have the greatest potential to be used as an ecologically friendlier alternative method for reducing the AF process.

## 2. Results

### 2.1. Native Marine Bacteria Involved in Biofilm Process

The bacterial adhesion tests allowed the selection of a collection of 11 native marine bacteria involved in the biofilm process in La Paz Bay. The strains isolated from sponges were closely related to the genera *Bacillus* and *Micrococcus*, while strains isolated from acrylic tiles immersed in the marina were mostly Gram negative bacteria closely related to *Flavobacterium*, *Pseudoalteromonas*, *Sagittula* and *Vibrio* (Table 1). The bacteria with more capacity of forming biofilm were *Bacillus pumilus* isolated from sponge *Aplysina gerardogreeni* and *Flavobacterium* sp. isolated from acrylic tile painted.

### 2.2. Toxicity

A toxicity test was performed on all extracts of algae and sponges obtained (see 4.2 for extracts list). Results are summarized in Table 2. After 24 h incubation, none of the macroalgae and sponge extracts affected in the survival rate and swimming behavior of *Artemia franciscana* (LC_50_ > 1000 µg mL^−1^) (Table 2). These results indicated that the extracts were non-toxic. When extracts were incorporated in experimental paints (see Section 4.5), the same results were obtained demonstrating the non-toxicity of the coating.

In contrast, the compound SDS (positive control) showed a toxic effect (LC_50_ = 14.01 µg mL^−1^) and the compounds TBTO and CuSO_4_ showed LC_50_ values of 8.53 and 6.21 µg mL^−1^, respectively (Table 2).

### 2.3. In Vitro Bioassays

#### 2.3.1. Antibacterial Bioassay

The activity of the extracts in the growth inhibition of marine bacteria involved in biofilm formation process was evaluated against the 11 native biofilm forming bacteria (Table 1), and the results were expressed as minimum inhibitory concentration (MIC) in μg mL^−1^ (Table 3). The most active extracts were: *Laurencia gardneri* (CH_2_Cl_2_), *Haliclona caerulea* (MeOH) and *Ircinia* sp. (MeOH); These 3 extracts led to growth inhibition of at least 4 strains with MIC values between 0.01 and 1 μg mL^−1^. It is of interest to note that the 11 selected bacteria display different susceptibilities toward the extracts tested: *Flavobacterium* sp., *Sagittula stellata*, *Micrococcus* sp. 1 and *Micrococcus* sp.3 showed the highest level of inhibition.

#### 2.3.2. Inhibition of Phenoloxidase Activity

**Figure 1:**
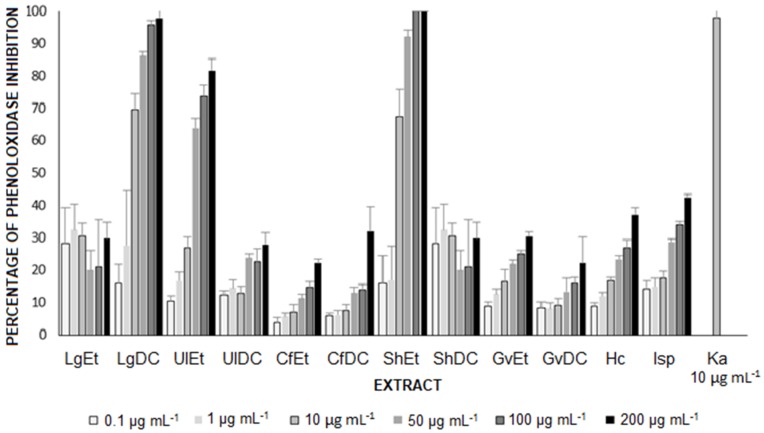
Effect of algae and sponge extracts (LgEt (*Laurencia gardneri* ethanol), LgDC (*Laurencia gardneri* CH_2_Cl_2_), UlEt (*Ulva lactuca* ethanol), UlDC (*Ulva lactuca* CH_2_Cl_2_), CfEt (*Codium fragile* ethanol), CfDC (*Codium fragile* CH_2_Cl_2_), ShEt (*Sargassum horridum* ethanol), ShDC (*Sargassum horridum* CH_2_Cl_2_), GvEt (*Gracilaria vermiculophylla* ethanol), GvDC (*Gracilaria vermiculophylla* CH_2_Cl_2_), Hc (*Haliclona caerulea)* and Isp (*Ircinia* sp.) and Ka (Kojic acid, used as a control) on the phenoloxidase inhibition.

The results of this study are presented Figure 1. The ethanolic extract of *Sargassum horridum* (Phaeophyceae), when assayed at 50 μg mL^−1^, displayed the highest inhibitory activity with 92% inhibition of enzymatic activity. These values were comparable to those obtained with kojic acid (Figure 1). The second most effective extract was the dichloromethane extract *Laurencia gardneri* (Rhodophyceae): when used at 100 μg mL^−1^, it led to 95% inhibition of the enzymatic activity. When sponge extracts were assayed, the phenoloxidase inhibition was inferior to 50%, even at the highest concentration tested.

#### 2.3.3. Inhibition Bacterial Adhesion Bioassay

According to the results obtained in the previous bioassays (see Section 2.3.1 and Section 2.3.2) and the absence of toxicity of the extracts, the most active extracts were selected for further investigation of their bioactivity as they appeared to be the best potential antifoulants. Thus extracts of *L. Gardneri* (CH_2_Cl_2_), *S. Horridum* (EtOH), *H. caerulea* (MeOH) and *Ircinia* sp. (MeOH) will be assayed toward the inhibition of bacterial adhesion and will be formulated in paint for field assay.

**Figure 2:**
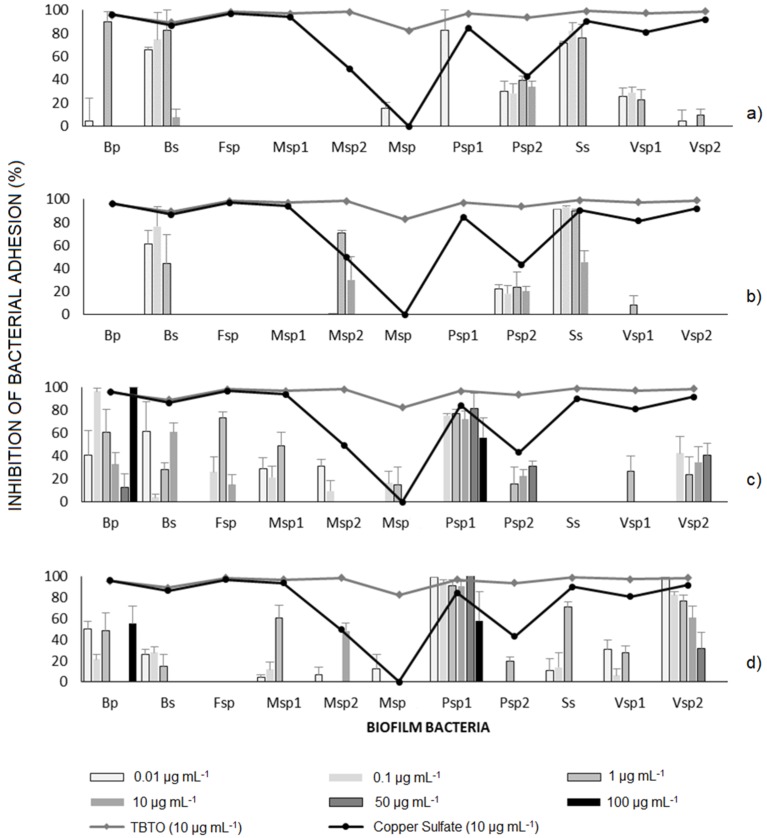
Potency of macroalgae and sponge extracts in the inhibition of bacterial adhesion (**a**) *Sargassum horridum* EtOH extract, (**b**) *Laurencia gardneri* CH_2_Cl_2_ extract, (**c**) *Haliclona caerulea* MeOH extract, and (**d**) *Ircinia* sp. MeOH extract.

The results of the inhibition of bacterial adhesion are presented in Figure 2. As a general pattern, macroalgae extracts led to high level of inhibition of *Sagittula stellata* (Ss) and *Bacillus subtilis* (Bs) (Figure 2a,b), while sponge extracts were not efficient toward these strains but instead inhibited the adhesion of *Vibrio* sp. 2 (Vsp2), *Pseudoalteromonas* sp. 1 (Psp1) and *Bacillus pumilus* (Bp) (Figure 2c,d). These strains were susceptible to the extracts and the results were comparable to the level of inhibition obtained with TBTO and CuSO_4_ demonstrating the high potency of the extracts.

All the extracts of algae showed a similar behavior and inhibited the adhesion of 3 bacteria: *Sagittula stellata* (Ss), *Pseudoalteromonas* sp. (Psp2) and *Bacillus subtilis* (Bs) at low concentrations (0.1 to 1 μg mL^−1^). The most susceptible strain was *Sagittula stellata*. (Ss), because it was possible to inhibit its adhesion over 80% (Figure 2a,b).

The extracts of the sponges were the most active and inhibited bacterial adhesion of most of the strains tested. The most susceptible strains were *Vibrio* sp. 2 (Vsp2), *Pseudoalteromonas* sp. 1 (Psp1), *Bacillus subtilis* (Bs) and *Bacillus pumilus* (Bp) (Figure 2c,d). The best result was obtained with sponge *H. caerulea* extract which inhibit the adhesion of most Gram positive and Gram negative strains (Figure 2c).

### 2.4. Antifouling Assay in Natural Conditions

**Figure 3:**
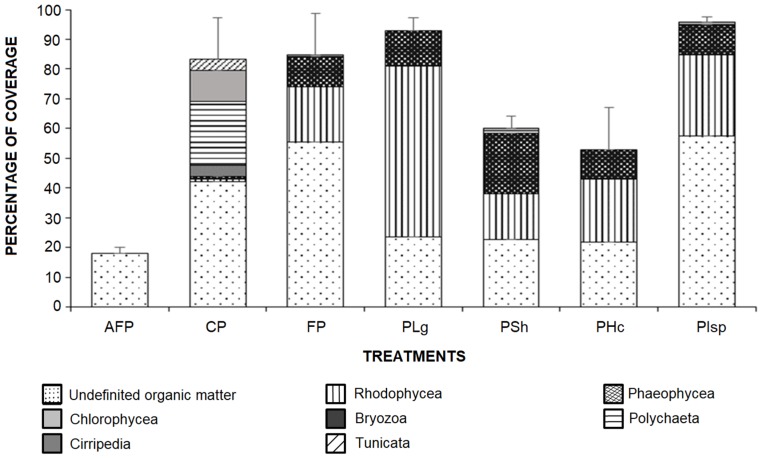
Epibiont percentage coverage of plates containing extracts after 40 days of exposure in the field (Marina La Paz). Antifouling Paint (AFP), Control plate without paint (CP), Formulated Paint (FP), *L. garneri extract* (PLg), *S. horridum* extract (PSh), *H. caerulea* extract (PHc)*, Ircinia* sp. extract (PIsp).

Field assay’s experiment results are presented in Figure 3. After 40 days immersion in La Paz Marina, the main colonizing organisms recorded on the control plate (CP—without paint) and the plate coated with formulated paint (FP) were Rhodophyceae (red macroalgae), Phaeophyceae (brown macroalgae), polychaetes (Polychaeta) and to lesser extent, barnacles (Cirripedia), bryozoans and tunicates. Meanwhile in the experimental plates containing algal or sponge extracts, the main colonizing organisms were only Rhodophyceae and Phaeophyceae, and some polychaetes (Figure 3).

The results highlighted that the experimental plates covered with paint + *H. caerulea* (PHc) and paint + *S. horridum* (PSh) were the best in term of antifouling activity: the percentage of coverage (52.7 and 60.1%, respectively) showed significant differences with respect to control plates (CP and FP) (Figure 3). In contrast, the paint with extracts of *Ircinia* sp. (PIsp) and *L. gardneri* (PLg) did not show antifouling activity and had more attachment than the CP (96 and 93%, respectively) (Figure 3). In all cases the experimental paints with extracts showed no toxicity at the concentration tested (Table 2).

**Figure 4:**
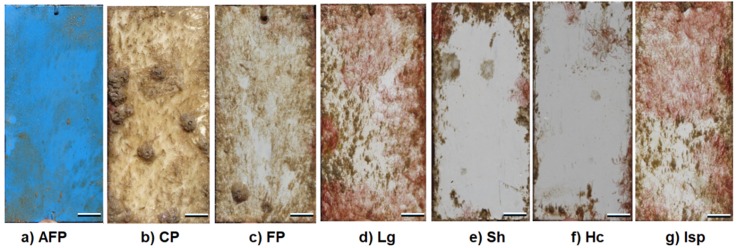
Pictures of plates (control, coated with commercial paint or extracts) after 40 days of immersion in the field (Marina La Paz). (**a**) Antifouling Paint (AFP), (**b**) Control plate without paint (CP), (**c**) Formulated Paint (FP), (**d**) *L. garneri extract* (PLg), (**e**) *S. horridum* extract (PSh), (**f**) *H. caerulea* extract (PHc) and (**g**) *Ircinia* sp. extract (PIsp) (Scale bar: 1 cm).

The comparison of CP with the treatments PHc and PSh demonstrated the effectiveness of the extracts as antifoulants, with inhibition of barnacle settlement. On most of the plates, an “edge-effect” was observed and thus the fixation of macroalgae occurred only in the edges of the plate (Figure 4d,g). After 40 days immersion of the plates in the marine La Paz, the control plate (CP) were fully covered by epibionts (Figure 4b), while the commercial antifouling paint (AFP) inhibited the settlement of macro-organisms was only covered in 18% of its surface by non-identifiable organic matter (Figure 4a).

## 3. Discussion

The problem of biofouling can be solved using a chemical ecology focused-approach, evaluating marine resources as possible antifouling agents against species from the same ecosystem. In this respect, algae and marine sponges have been studied in the search for compounds that inhibit any of the stages of biofouling [19,20]. In the marine environment, the settlement of macrofoulers depends significantly on the biofilm establishment [21]. To avoid growth of fouling organisms on marine structures, it is necessary to control the proliferation of adhesive microbes [22].

The activity of the majority of commercial antifouling products corresponds to international activity criteria: thus, it is required to have a broad spectrum activity, and on specific target species such as *Ulva intestinalis* or *Balanus amphitrite*. The same applies to toxicity tests, which are carried out on a precise list of species. Nevertheless, when a paint formulation is developed for use in a limited geographical area, such tests are sometimes very unrepresentative of the ecosystem and therefore of local fouling pressure [10]; we then find ourselves with poorly adapted formulations and this can lead to a decrease in efficiency and an increased risk of environmental toxicity.

Marine algae and sponges from various regions have been evaluated as a source of metabolites with antifouling activity. It was concluded that these organisms represent good sources of antifoulants due to the presence of a great diversity of chemical compounds with diverse and novel structures. These compounds have an ecological function as a chemical defense against other organisms to avoid the settlement of bacteria and epibiont organisms [23]. In this type of research program, it may seem repetitive to evaluate again a genus, such as *Laurencia*, from which fractions and compounds have been reported that inhibit the growth of biofouling-forming bacteria and the settlement of barnacles [24,25,26,27]. However, a recent study has demonstrated that results from laboratory assays did not fully concur with the AF activity of the paints in the field trial, and because of the lack of field tests, the results of in vitro bioassays are not enough to be critically discussed [17]. Furthermore, the production of specific compounds by organisms in their ecological context can potentially have a better efficacy on target species when sampling and testing organisms from the same environment [10]. In this sense, the aim of this study is to evaluate the AF potential of marine benthic resources from Baja California Sur in a more integrated manner, both with various laboratory bioassays with native biofouling-forming bacteria, as well as in field tests against macro-fouling.

### 3.1. Native Marine Bacteria Involved in Biofilm Process

The bacterial adhesion tests allowed the selection of 11 native bacteria involved in the biofilm process in the bay of La Paz. The strains were closely related to the genera *Bacillus*, *Micrococcus*, *Flavobacterium*, *Pseudoalteromonas*, *Sagittula* and *Vibrio* (Table 1). These bacteria are considered to be pioneers in the process of colonization and have great participation in the production of biofilms [22,28,29,30,31].

*Bacillus pumilus* and *Flavobacterium* sp. showed the higher adhesion power. The genus *Bacillus* has the capacity to produce endospores and possesses a swarming motility that facilitated the formation of the biofilm and its persistence [32,33]. Specifically, for *B. pumilus*, the degree of adherence in the biofilm can be variable from intermediate level [34] to strong attachment [11]. In the case of the *Flavobacterium* genus, the strong biofilm production is regulated by active expression of several putative adhesins [35].

The isolates from the acrylic tiles immersed in the marina were mostly Gram negative bacteria and only one Gram positive bacteria (Table 1). Isolates from the marine environment in other regions showed the same trend, where the group of Gram-negative bacteria had higher percentages [36]. Conversely, in previous research in La Paz Bay, isolated biofilm-forming bacteria were only Gram positive bacteria [11]. The communities are strongly influenced by environmental conditions and geographic location [37] and these contrasts show the importance of identify the biofilm-forming bacteria and know how are affected by the AF agents under evaluation.

### 3.2. Toxicity

The environmental impacts of commercial antifouling paints demonstrate the need to develop new strategies that are truly non-toxic and effective. The International Maritime Organization (IMO) prohibited the use of TBT in antifouling paints due to them causing serious damage in the marine environment, but in the case of cuprous oxide there is still use despite its toxicity and negative effect on the development of some marine organisms [38]. One of the most important criteria currently taken into account in marketing authorization applications is the results of the toxicity assessment. This is why in this research program, we prioritize an ethical methodology and we have decided to evaluate this parameter very early on (by laboratory tests) in order to work only on non-toxic extracts, especially during field tests.

None of the macroalgae and sponge extracts evaluated in our study did show a toxic effect against *Artemia franciscana* (LC_50_ > 1000 µg mL^−1^). According to the classification of Fernández-Calienes et al. [39], the toxicity ranges are categorized as extremely toxic (LC_50_ < 10 μg mL^−1^), very toxic (10 < LC_50_ < 100), moderately toxic (100 < LC_50_ < 1000) and non-toxic (LC_50_ > 1000 μg mL^−1^). Therefore, all extracts can be considered to be non-toxic. In contrast, the compound SDS (positive control) was very toxic (LC_50_ = 14.01 µg mL^−1^) and the compounds like TBTO and CuSO_4_ that are considered extremely toxic [40,41], showed LC_50_ values of 8.53 and 6.21 µg mL^−1^, respectively (Table 2). It is important to evaluate the toxicity before starting with antifouling activity tests. Moderate or no toxicity is an important factor when proposing candidates for AF agents [42].

### 3.3. In Vitro Bioassays

#### 3.3.1. Antibacterial Bioassay

Macroalgae are considered a good resource in the search of AF agents. Screening programs indicated that the Phyla Rhodophyta is the most promising in the isolation of novel AF compounds [43]. The *Laurencia* genus has been widely studied, and a large number of metabolites with antifouling activity have been isolated like elatol, omaezallene and 2,10-dibromo-3-chloro-7-chamigrene. However, this does not discourage the search for new compounds, and recently 6 new antifouling compounds have been described as omaezol, intricatriol, hachijojimallenes A and B, debromoaplysinal and 11,12-dihydro-3-hydroxyretinol [27]. The bioactivity of *Laurencia gardneri* collected in Baja California Sur, Mexico has been only poorly studied so far. Crude ethanol extracts of this species were not active against human pathogenic bacteria, [44]. However, in our work, the extract of the same species with a different solvent (CH_2_CL_2_) was one of the most active against bacteria involved in biofouling process. The extracts of *Laurencia johnstonii* harvested from the Gulf of California showed antifouling activity against bacteria and microalgae [26]. Therefore, it is important to continue evaluating new resources [43], especially against native strains involved in the biofouling process.

Sponges have an extensive record of inhibiting the growth of adherent bacterial strains. In its natural environment, the inhibition of epibiosis benefits the survey and longevity of the organism [45]. In this sense, species of the *Haliclona* genus were evaluated in antifouling activity and showed that the extracts are good antifouling agents in the inhibition of the growth of fouling bacteria [46,47,48,49].

#### 3.3.2. Inhibition of Phenoloxidase Activity

For inhibiting the attachment of fouling organisms, a strategy is the search for direct enzyme inhibitors of phenoloxidase (enzyme involved in the attachment of mussel and barnacles) [50]. The method based on the measurement of phenoloxidase activity in the presence of different substances with antifouling activity development in vitro has the advantage of being a specific, repeatable, sensitive and rapid bioassay [51]. Several inhibitors of phenoloxidase have been isolated from natural sources, like quercetin and kojic acid [52,53]. Brown algae are an important source of metabolites derived from Phloroglucinol that showed a decrease in the enzymatic activity of the phenoloxidase. The results of this study showed that the ethanolic extract of the brown algae *S. horridum* had the best activity at 50 μg mL^−1^ (92% inhibition the enzyme). These values were similar to those obtained with Kojic acid (Figure 1) and thus, this has a high interest for paint formulation. The second most effective extract was dichloromethane from red algae *L. Gardneri* (CH_2_Cl_2_), achieving an inhibition of the phenoloxidase at 95% when used at 100 μg mL^−1^ (Figure 1). In red algae, bromated phenolic and other polyphenolic compounds are as well-known inhibitors of phenoloxidase [54,55,56].

The high number of compounds isolated from marine sponges and compounds that inhibited the phenoloxidase activity, like Hemibastadin, isolated from the *Iantella* genus, stimulate the search of new antifouling compounds with this type of mode of action [50]. In the present research, the percentage of enzymatic production in the sponge *Ircinia* sp. and *Haliclona caerulea* was lower of 50%, even at the highest evaluated concentration. There are no previous records for these species, but it has been observed that extracts of *Haliclona* sp. cause a negative stimulus in the adherence of the blue mussel at concentrations of 100 μg mL^−1^ [57].

#### 3.3.3. Inhibition Bacterial Adhesion Bioassay

The results showed that the extracts that inhibited the adhesion bacterial were not able to inhibit bacterial growth of some strains. It is appreciated that the extract was selective in the adhesion inhibition. The best result was obtained with sponge *H. caerulea* extracts (Figure 2c). Avoiding the bacterial adhesion on surfaces is crucial in antifouling strategies. Compounds with antibacterial activity cause the death of microorganisms, but these can easily accumulate on surfaces, leading to bacterial contamination and biofilm formation and increase the risk of selecting for resistant bacteria that can then spread in the environment [18].

### 3.4. Antifouling Assay in Natural Conditions

The formulated paint (FP) remained in good condition on the plate over time and thus it was determined that it represents a good base to evaluate the extracts. The comparison of negative control (FP) and control (CP) with the treatments PHc and PSh indicates the effectiveness of the *H. caerulea* and *S. horridum* extracts, which inhibited the settlement of barnacles, and it is evident that the fixation of macroalgae occurred only in the edges of the plate (Figure 4d,g).

In the seawater, any unprotected immersed substratum is rapidly colonized by marine organisms [17]. In the marina of La Paz, after 40 days of immersion, the plates without any type of paint (CP) were covered by most epibiont organisms (Figure 4b), while the commercial antifouling paint (AFP) inhibited the settlement of all organisms and was only covered in 18% of non-identifiable organic matter (Figure 4a). The coatings with *Sargassum* and *Haliclona* extracts can be good AF agents. Although there is no previous research where *Sargassum* or *Halicona* extracts have been incorporated in paint to evaluate their effectiveness in the field, different studies have referred to the genus *Sargassum* possessing excellent characteristics and compounds like palmitic acid that presented antifouling and non-cytotoxic properties [58] to be used in the formulation of antifouling materials, such as paints. Sponges of the genus *Haliclona* have been a good source of bioactive compounds, and some of these, like haliclona ciclamine A and halaminol A, have activity preventing fouling and colonisation, even inducing rapid larval settlement but preventing subsequent metamorphosis at precisely the same stage [48]. Another alkaloid, isolated from the marine sponge *H. exigua*, is bis-1-oxaquinolizidine, considered to be an environmentally compatible antifoulant against barnacle cyprids and/or antimicrobial agent against the growth of fouling bacteria [47]. Different species of *Haliclona* that differ in chemical composition have similar effects in preventing the colonization of invertebrate larvae and supporting the proposal that multiple chemical solutions are available to solve a single ecological problem [48,49]. When using extracts in paint formulation, the advantage is that mixtures of molecules are incorporated, thus allowing synergistic activities. This type of approach makes it possible to copy the natural strategies of organisms and to obtain the totality of the diversity of defense molecules produced by an organism.

In the search of antifouling agents, it is important to perform assays in the laboratory and field because only few extracts or compounds are incorporated in paint and put into marine experiments [16], and the results in laboratory assays do not always provide sufficient information with respect to the field performance of chemically-active paints [17]. For this research, when comparing the results observed specificity in the activity in the laboratory and field assays, per example, in the case of extracts of *L. gardneri* (CH_2_Cl_2_) and *Ircinia* sp. laboratory’s results were promising, but when tested in real conditions of immersion in the marina, they led to promotion of the biofouling settlement. This result is difficult to interpret, and it is necessary to evaluate in future studies the different factors (biotic and abiotic) that help explain this phenomenon. In the case of the other 2 species, *Sargassum horridum* extract (EtOH) only was able to inhibit the growth and adhesion of some bacteria but had the best results in the inhibition of phenoloxidase, while the opposite behavior was observed with the extract of *H. caerulea.* However, in the field test, the results are similar, and these extracts are a good resource to inhibit the fouling attachment.

The use of extracts incorporated in non-toxic paint could represent a more economical and viable option, if the organism abundance is enough to supply the resource. In the case of macroalgae *S. horridum*, it has potential because this genus has worldwide distribution and is abundant in different regions [59,60]. In the Gulf of California, the *Sargassum* beds covered a surface area of 4.22 km^2^ with an estimated standing crop of 19,206 t and until now these beds are not exploited for commercial purposes [61]. In the last years, due to the climate changes, a model to assess the changes in macroalgae beds in Europe, predicted the expansion of the range of distribution of *Sargassum* species [62]. Furthermore, large pelagic *Sargassum* uplands are increasing in different coasts causing significant environmental and economic damage [63]. Different use potentials are described [64] and the use in the formulation of AF paints may be possible, but it is necessary to study economic feasibility to determine a commercial development.

## 4. Materials and Methods

### 4.1. Sample Collection

Five species of macroalgae were collected from the seashore by hand picking in San Juan de la Costa locality (24°22′15″ N–110°41′22″ W): *Codium fragile* (Suringar) Hariot 1889, *Ulva lactuca* Linnaeus, C. (1753), *Sargassum horridum* Setchell & N.L.Gardner (1924), *Laurencia gardneri* Hollenberg (1943) and *Gracilaria vermiculophylla* (Ohmi) Papenfuss (1967).

Two species of sponges, *Ircinia* sp. Nardo (1833) and *Haliclona caerulea* Hechtel (1965), were collected by diving at a 1.5 m depth in Pichilingue locality (24°17′ N–110° 20′ W).

Immediately after collection, specimens were placed into plastic bags, transported on ice to the laboratory within 2 h and then were cleaned to remove salt and epiphytes organisms. The macroalgae were sun-dried, ground into fine particles and stored in bags. The sponges were lyophilised and then stored at −20 °C until processed.

### 4.2. Preparation of Extracts

For macroalgae, 30 g of dry material of each species was macerated in dichloromethane (300 mL) at room temperature during 48 h. The solution was then separated from the algae residue by filtration with qualitative filter paper (Whatman Grade 1). This extraction was repeated 3 more times. The solutions obtained from each filtration were concentrated in a rotary evaporator at 38 °C to obtain dichloromethane extracts (CH_2_Cl_2_). Subsequently, the algae residue was extracted with ethanol 80% (300 mL), 4 times following the same protocol as above to obtain ethanolic extracts (EtOH) [65].

For sponges, the lyophilized material (50 g) was macerated into a mixture of acetone/methanol 1:1 (300 mL) over 2 h. and then hand ground in a mortar. The solution was filtered and concentrated in a rotary evaporator at 38 °C. The solid was dissolved with methanol 3 times, and the solution was joined and concentrated to obtain sponge methanolic extracts (MeOH). All extracts were stored at −20 °C until use [66].

### 4.3. Isolation of Native Marine Bacteria Involved in Biofilm Process

The test strains were obtained using 2 approaches:

(1) Strains associated to sponges (*Aplysina gerardogreeni* Gomez & Bakus 1992 and *Mycale ramulosa* Carballo & Cruz-Barrazo 2010) and involved in biofilm formation were targeted. Thus, 7 associated sponge-bacteria belonging to *Bacillus* and *Micrococcus* genus collected in a previous study were evaluated for their capacity to form biofilm. This was done by standard colorimetric assay using crystal violet [67].

(2) Bacteria involved in the biofouling process on man-made surfaces were collected and isolated from the Marina of La Paz, B.C.S., Mexico (24°08′32″ N–110°18′39″ W): 4 sandblasted acrylic tiles (4 × 12 cm) were immersed in the Marina at 2 m depth (February). Two acrylic tiles were coated with formulated paint (FP) and 2 tiles were without paint (WP). After 48 h immersion, the tiles were removed, placed in sterile plastic bags and transported immediately to the laboratory. The isolation of bacterial strain was performed in sterile conditions. The tiles were rinsed with sterile seawater (SSW) and rubbed with a swab and placed in 10 mL of SSW with the aim to obtain a bacterial suspension. Serial dilution (10^−1^ to 10^−5^) of the suspension, were prepared and 100 µL aliquots were spread on plates of Difco Marine Agar in triplicate. The bacterial colonies were isolated and characterized based on morphology and Gram staining. The capacity of bacteria for biofilm formation was evaluated by colorimetric assay with crystal violet [67].

The bacteria involved in biofilm formation and used in the antifouling assays were identified by partial sequencing of 16S rRNA gene fragments. The DNA was obtained by the modified method of Sambrook and Russell [68] and Ausubel et al. (2002) [69]. PCR amplicons were purified, and DNA was sequenced by MACROGEN (Korea). The search in the Genbank database was performed using the BLAST program at the National Centre for Biotechnology Information website (http://www.ncbi.nlm.nih.gov/BLAST/), and sequencing data were analyzed by comparing the sequences of nearest relatives found by BLAST searching.

### 4.4. Assays in Laboratory

#### 4.4.1. Toxicity Assay

The toxicity of extracts was evaluated using the brine shrimp lethality assay [70]. Experiments were run in 96-well flat bottom microplates (Costar 3596). 100 µL of extracts (dissolved in methanol) at 1, 10, 100 and 1000 µg mL^−1^, in 6 replicates, were added to the wells and let to evaporate (over 12 h in an extraction hood or until total evaporation of the methanol). Then a suspension of SSW (200 µL), containing 10 nauplii of brine shrimp (*Artemia franciscana* Kellogg 1906) previously hatched, was added to each well and incubated at 30 °C for 24 h. Positive controls consisted of Tributyltin oxide (TBTO) solutions at 6, 9, 12 and 15 µg mL^−1^ and wells with only methanol evaporated were used as negative control. The number of survivors in each well was counted under a stereoscopic microscope, and percentage of death was calculated. The lethal concentration at 50% (LC_50_) was calculated by Probit Test in the Stat Graphics program.

#### 4.4.2. Antibacterial Bioassay

Stock solutions of extracts were prepared in methanol (as a carrier solvent) with final concentrations of 0.01, 0.1, 1, 10 and 50 and 100 μg mL^−1^. An aliquot (100 μL) of the extracts solutions was pipetted into 96-well plates. The solvent was evaporated and then the plates were sterilized in a UV sterilization cabinet for 30 min [61]. The assay was performed against the native bacteria involved in biofouling (*Bacillus pumilus, B. subtilis, Flavobacterium* sp. *Micrococcus* sp. 1, *Micrococcus* sp. 2, *Flavobacterium* sp., *Pseudoalteromonas* sp. 1, *Pseudoalteromonas* sp. 2, *Sagittula stellata, Vibrio* sp. 1 and *Vibrio* sp. 2) The bacterial cultures were grown overnight on marine agar at 35 °C and adjusted according to Martín-Rodríguez et al. [71] with a density of 1 × 10^8^ cell mL^−1^. The plates were inoculated with 200 μL of bacterial cell suspension and incubated for 48 h at 35 °C. The plates were read at 620 nm in a microplate reader, and the results were expressed as minimum concentration inhibitory (MIC).

#### 4.4.3. Inhibition of Phenoloxidase Activity

The effect of the extracts as inhibitors of the phenoloxidase (EC1.14.18.1 Sigma), an enzyme involved in the settlement of the bivalve mollusks, was evaluated using the protocol of Hellio et al. (2015) [67]. The extracts were dissolved in DMSO and serially diluted with a potassium phosphate buffer (50 mM, pH 6.5) to obtain concentrations of 0.1, 1, 10, 50, 100 and 200 μg mL^−1^. In 96-well plates, 70 μL of extracts at different concentrations were added to 30 μL of enzyme (diluted in phosphate buffer at 333 U mL^−1^) and incubated 5 min at 25 °C. L-Dopa 10 mM in 50 mM phosphate buffer (pH 6.8) was added and used as substrate. The plates were incubated 30 min at 30 °C. The absorbance was recorded at 450 nm with 10 lectures every 30 s. Positive controls were kojic acid at 1, 10, 50, 100, 200 and 400 μg mL^−1^ and TBTO a 10 μg mL^−1^, and negative control was only buffer. The test was realized in triplicate. The results are expressed as the inhibition percentage of phenoloxidase activity with respect to negative control.

#### 4.4.4. Inhibition Bacterial Adhesion Bioassay

According to the results of the aforementioned tests, only the most 4 active extracts in the inhibition of bacterial growth and inhibition of Phenoloxidase activity were selected to continue with the study. The assay of bacterial adhesion was performed in accordance with Leroy et al. [72]. We used this method to evaluate the inhibition of adherent marine bacteria by direct quantification. An aliquot (100 μL) of the extract solutions was pipetted into black 96-well plates with flat bottoms. The solvent was evaporated, and then the plates were sterilized in a UV sterilization cabinet for 30 min. The bacteria involved in biofouling, was grown overnight on marine agar and adjusted to an optical density of 1.0 at 585 nm. Then, 100 μL of the bacterial suspension was inoculated in each well using a multichannel pipette. The covered plate was incubated for 48 h at 35°C using horizontal shaking (150 rev min^−1^). The non-adhered bacteria were eliminated by 3 washings with NaCl solution (36 g·L^−1^), and the adhered bacteria were fixed with a sterile solution of NaCl solution (36 g·L^−1^) containing formaldehyde (2.5%) for 1.5 h at 4 °C. The plate was washed with sterile NaCl solution and dried at room temperature. In dark conditions, the adherent bacteria were stained with 100 μL of 4’6-diamidino 2-fenilindol (DAPI) at 4 μg mL^−1^ for 20 min. Then, 3 washings with NaCl solution (36 g L^−1^) were realized. DAPI was solubilized in 100 μL of an ethanol solution (95%), then shaken for 15 min. Fluorescence was measured at 350 nm excitation and 510 nm emission wavelengths in a microplate fluorescence reader (TECAN). TBTO and copper sulphate were utilized as control. All assays were realized by 6 replicates. The results were expressed as inhibition bacterial adhesion percentage.

### 4.5. Antifouling Assay in Natural Conditions

The four more active extracts in the laboratory assay were used for the field test. The antifouling paint matrix was prepared in laboratory conditions in accordance with Acevedo et al. [16]. The paint composition was colophony (27%), oleic acid (6%), xylene (20%), white spirit (20%), zinc oxide (16.2%) and calcium carbonate (10.8%). The paint was filtered and separated into 5 parts for different treatment, and 1 part with methanol was used as a negative control (FP). The other paint parts were used to include the different organic extracts (0.1% *w*/*w*) previously dissolved in methanol: *S*. *horridum* (PSh), *L. gardneri* (PLg), *Ircinia* sp. (PIsp) and *H. caerulea* (PHc).

Sandblasted acrylic tiles (4 × 12 cm), previously degreased, were covered with 4 coats of paint and allowed to dry for 24 h between each application. The tiles were placed in a PVC frame. Additionally, in the frame, other control was included: acrylic unpainted plates (CP) and plates covered with commercial antifouling paint (AFP) as reference. All treatments were run in triplicate. The frame with plates was immersed in a Marina La Paz in La Paz, Baja California Sur, México (24°09′18.6″ N–110°19′31.7″ W) at a 2 m depth for 40 days.

### 4.6. Coverage Percentage of Epibionts

Percentages of coverage of each group of organisms in each plate were estimated after 40 days of exposure in the sea water, using a dot grid method [16] and CPCe program. The coverage data refer to the percentage of the area colonized by each group of organisms on the plate exposed to light. The differences between each treatment were determined by a one-way analysis of variance test (ANOVA).

## 5. Conclusions

In the search of an ecologically friendlier alternative for reducing the AF process, it is necessary use more efficient approaches, therefore in this work the combined in vitro and marine field assays helped to highlight antifouling agents from macroalgae and sponge resources collected in the Gulf of California. The methodology used followed the next steps: a) isolate and cultivate key bacteria involved in biofilm formation, b) select, in the same ecosystem, organisms with low epibiosis and produce extracts; c) evaluate the toxicity of the extracts, d) evaluate in laboratory assays the capacity to inhibit both growth and adhesion bacteria involved in biofilm and the activity of phenoloxidase, and e) the most promising extracts must be included in experimental painting matrix and immersed in the sea. From laboratory-based assays, we concluded that the most active extracts (towards biofilm-forming bacteria and phenoloxidase) were *L. gardneri* (CH_2_Cl_2_), *S. horridum* (EtOH), *H. caerulea* and *Ircinia* sp. (MeOH). The inclusion of these extracts in paint showed that the results from lab-based assays were not always in concordance with field results. Thus, when immersed in the marina, *L. gardneri* (CH_2_Cl_2_) *and Ircinia* sp. promoted the biofouling settlement; however, the paint formulated with *S. horridum* (EtOH) and *H. Caerulea* were good antifouling agents as they inhibited the attachment of algae and barnacles and these formulations were non-toxic. The biomimetic approach for the development of new antifouling solutions demonstrate that when incorporating extracts from marine organisms, it is possible to reach promising activity toward the inhibition of fouling from the same geographical area. The use of this approach in other zones can lead a more comprehensive solution of controlling biofouling on man-made surfaces.

## Figures and Tables

**Table 1:** Native biofilm forming marine bacteria from coasts of Baja California Sur selected for being used in antibacterial and anti-adherent activity assays.

ID Strain	Isolated Origin	Closely Related Species	Access Number GenBank	% Identity	Capacity for Forming Biofilm *
Ap-20	SAp	*Bacillus pumilus* (Bp)	JF501099.1	98	++++
Ap-167	SAp	*Bacillus subtilis* (Bs)	JX215561.1	100	+++
My-20	SMr	*Micrococcus* sp. 1 (Msp1)	FR750272.1	98	+++
My-27	SMr	*Micrococcus* sp. 2 (Msp2)	HQ188562.1	99	+++
MLP-Sp20	WP	*Micrococcus* sp. (Msp)	KX082870.1	99	+++
MLP-Sp21	FP	*Flavobacterium* sp. (Fsp)	KF2823252.1	99	++++
MLP-Cp11	FP	*Pseudoalteromonas* sp. 2 (Psp2)	KX233660.1	100	+
MLP-Sp7	WP	*Pseudoalteromonas* sp. 1 (Psp1)	KM041227.1	100	+++
MLP-Cp5	FP	*Sagittula stellata* (Ss)	HG315014.1	98	++
MLP-Sp3	WP	*Vibrio* sp. 1 (Vsp1)	KR075009.1	100	+++
MLP-Sp6	WP	*Vibrio* sp. 2 (Vsp 2)	DQ146972.1	99	+++

Isolation origin: SAp (sponge *Aplysina gerardogreeni*), SMr (sponge *Mycale ramulosa*), WP (Acrylic tile Without Paint), FP (Acrylic tile with Formulated Paint). * (OD 595 nm), + (0.25–0.49), ++ (0.5–0.74), +++ (0.75–0.99), ++++ (>1.00).

**Table 2:** Determination of LC_50_ values for macroalgae and sponge extracts on shrimp *Artemia franciscana* nauplii.

Species	Extract	LC_50_(µg mL^−1^)
Macroalgae		
	EtOH	>1000
*L. gardneri*	CH_2_Cl_2_	>1000
	CH_2_Cl_2_/Paint	>1000
*U. lactuca*	EtOH	>1000
	CH_2_Cl_2_	>1000
*C. fragile*	EtOH	>1000
	CH_2_Cl_2_	>1000
	EtOH	>1000
*S. horridum*	EtOH/Paint	>1000
	CH_2_Cl_2_	>1000
*G. vermiculophyla*	EtOH	>1000
	CH_2_Cl_2_	>1000
Sponges		
*H. caerulea*	MeOH	>1000
	MeOH/Paint	>1000
*Ircinia * sp.	MeOH	>1000
	MeOH/Paint	>1000
Controls		
SDS		14.01
TBTO		8.73
CuSO_4_		6.21

**Table 3:** Minimum inhibitory concentration (MIC) of macroalgae and sponge extracts on marine bacteria involved in biofilm formation.

Organism	Extract	Antibacterial Activity (MIC Values in µg mL^−1^)										
		Bp	Bs	Fsp	Msp1	Msp2	Msp	Psp1	Psp2	Ss	Vsp1	Vsp2
Macroalgae												
*L. gardneri*	EtOH	1	>100	0.01	0.01	>100	0.1	>100	100	0.01	>100	>100
	CH_2_Cl_2_	1	1	0.01	0.01	>100	0.1	>100	>100	1	>100	>100
*U. lactuca*	EtOH	>100	>100	0.01	50	>100	1	>100	>100	>100	>100	>100
	CH_2_Cl_2_	>100	>100	0.01	0.01	>100	0.01	>100	>100	100	>100	>100
*C. fragile*	EtOH	>100	>100	0.01	0.01	>100	0.1	>100	50	10	>100	>100
	CH_2_Cl_2_	>100	>100	0.01	0.01	>100	1	>100	>100	100	>100	>100
*S. horridum*	EtOH	10	>100	0.01	10	>100	0.1	>100	50	50	>100	>100
	CH_2_Cl_2_	>100	>100	0.01	>100	>100	1	>100	>100	>100	>100	>100
*G. vermiculophylla*	EtOH	>100	>100	0.01	10	>100	>100	>100	>100	100	>100	>100
	CH_2_Cl_2_	>100	>100	0.01	0.01	>100	0.01	>100	1	>100	>100	>100
Sponges												
*H. caerulea*	MeOH	0.1	>100	0.01	1	>100	0.1	>100	>100	0.1	>100	>100
*Ircinia* sp.	MeOH	10	>100	0.01	0.01	>100	0.01	>100	>100	0.1	>100	>100

Bp (*Bacillus pumilus*), Bs (*Bacillus subtilis*), Fsp (*Flavobacterium* sp.), Msp1 (*Micrococcus* sp. 1), Msp2 (*Micrococcus* sp. 2), Msp (*Micrococcus* sp.), Psp1 (*Pseudoalteromonas* sp. 1), Psp 2 (*Pseudoalteromonas* sp. 2), *Ss* (*Sagittula stellata*), Vsp1 (*Vibrio* sp. 1), Vsp2 (*Vibrio* sp. 2).

## Abbreviations

AF	Antifouling
Bp	*Bacillus pumilus*
Bs	*Bacillus subtilis*
CfEt	*Codium fragile* ethanol extract
CfDC	*Codium fragile* ethanol extract
DMSO	Dimethyl Sulfoxide
Fsp	*Flavobacterium* sp.
GvEt	*Gracilaria vermiculophylla* ethanol extract
GvDC	*Gracilaria vermiculophylla* dicloromethane extract
Hc	*Haliclona caerulea*
Isp	*Ircinia* sp.
IMO	International Maritime Organization
Ka	Kojic acid
LgEt	*Laurencia gardneri* ethanol extract
LgDC	*Laurencia gardneri* dicloromethane extract
Msp	*Micrococcus* sp.
Msp1	*Micrococcus* sp. 1
Msp2	*Micrococcus* sp. 2
Psp1	*Pseudoalteromonas* sp. 1
Psp2	*Pseudoalteromonas* sp. 2
ShEt	*Sargassum horridum* ethanol extract
ShDC	*Sargassum horridum* dicloromethane extract
SDS	Sodium Dodecyl Sulfate
SSW	Sterile Sea Water
*Ss*	*Sagittula stellata*
TBT	Tributyltin
TBTO	Tributyltin Oxide
UlEt	*Ulva lactuca* ethanol extract
UlDC	*Ulva lactuca* dicloromethane extract
Vsp1	*Vibrio* sp. 1
Vsp2	*Vibrio* sp. 2
